# The Value of Patch-Choice Copying in Fruit Flies

**DOI:** 10.1371/journal.pone.0112381

**Published:** 2014-11-06

**Authors:** Shane Golden, Reuven Dukas

**Affiliations:** Animal Behaviour Group, Department of Psychology, Neuroscience & Behaviour, McMaster University, Hamilton, Ontario, Canada; Utrecht University, Netherlands

## Abstract

Many animals copy the choices of others but the functional and mechanistic explanations for copying are still not fully resolved. We relied on novel behavioral protocols to quantify the value of patch-choice copying in fruit flies. In a titration experiment, we quantified how much nutritional value females were willing to trade for laying eggs on patches already occupied by larvae (social patches). Females were highly sensitive to nutritional quality, which was positively associated with their offspring success. Females, however, perceived social, low-nutrition patches (33% of the nutrients) as equally valuable as non-social, high-nutrition ones (100% of the nutrients). In follow-up experiments, we could not, however, either find informational benefits from copying others or detect what females' offspring may gain from developing with older larvae. Because patch-choice copying in fruit flies is a robust phenomenon in spite of potential costs due to competition, we suggest that it is beneficial in natural settings, where fruit flies encounter complex dynamics of microbial communities, which include, in addition to the preferred yeast species they feed on, numerous harmful fungi and bacteria. We suggest that microbial ecology underlies many cases of copying in nature.

## Introduction

In many animal species, individuals copy the choices of others. Examples include choices of feeding sites [1]–[3], territories [4], [5], egg laying substrates [6]–[8] and mates [9]–[11]. Depending on the system, copying can have substantial effects on organismal ecology and evolution. For example, conspecific aggregation at feeding and egg laying sites can promote species coexistence [12], [13] and mate choice copying can influence the intensity and direction of sexual selection [14]–[16].

While it is widely agreed that copying can influence animal ecology and evolution, it is often unclear how the possible fitness benefits from copying outweigh the likely costs. For example, patch-choice copying typically involves a focal individual choosing a feeding or egg laying site that is either occupied by other individuals (models) or contains products left by these individuals. There are probably only two non-mutually exclusive explanations for such copying. The first explanation involves pure information: a focal can either find a satisfactory patch faster, or locate a better patch among the available alternatives by copying others than by exploring on its own [4], [17]–[19]. That is, the first explanation focuses on two related difficulties that animals have in locating optimal resource patches. Either the patches are hidden, so it takes time to find them, or it is difficult and time consuming to assess the multitude of features that determine patch quality. Given individuals' limited time horizon, focals that copy others can shorten the time devoted to exploration and hence increase the time spent exploiting without compromising on the quality of the patch utilized. This proposition, of course, is based on the tenuous assumption that the models indeed have chosen the optimal patch.

The other explanation for patch-choice copying involves material benefits that focals can gain from joining others, which include reduced per capita risk of attack by predators and parasitoids, and enhanced foraging efficiency and thermoregulation [20]–[25]. It is worth noting that, when patch-choice copying involves joining others, focals and models might face asymmetric payoffs: while a focal can gain more from joining than from settling alone, the models might lose from having another individual joining [26]. The obvious costs from joining others are competition for resources and reduced patch quality caused by accumulating waste products [7], [19], [20]. Competition can cause another possible asymmetric payoff that is size dependent. For example, newly hatched larvae may lose more from competition than the older resident larvae.

While there are numerous reports of copying in a wide variety of species and contexts, the value of copying has been rarely quantified. We have recently developed protocols for quantifying patch-choice copying in fruit flies (*Drosophila melanogaster*). Larvae and adults from both established laboratory strains and recently caught wild populations copy the choices of others: adult females prefer the egg laying substrates chosen by other females [27], [28], both male and female adults are attracted to volatiles emanating from conspecific larvae, females show a strong preference for laying eggs in patches with larvae over unoccupied alternatives, and larvae also show significant attraction to patches already occupied by larvae [29]–[31]. The establishment of fruit flies as a model system for research on patch-choice copying offers new opportunities. First, the fruit fly system allows one to conduct highly controlled experiments assessing the factors that influence patch choice copying. Second, findings from the behavioral analyses of patch-choice copying can be extended to research on the genetics and neurobiology of such behavior in a highly amenable model system. Indeed there has recently been increased interest in establishing simple model systems for research on the mechanisms that control social behavior as well as behavioral decisions in general [32]–[35].

To elucidate the value of patch-choice copying in fruit flies, we conducted a series of experiments. We began with a titration experiment designed to quantify the perceived value that females assign to food occupied by larvae. This involved testing female preferences between reference patches and test patches of varying food qualities, which were either occupied or unoccupied by larvae. In follow-up experiments, we compared larval success on occupied and unoccupied patches of relevant food qualities. This allowed us to translate patch-choice copying by females into the consequent success of their offspring. Because females showed strong patch-choice copying even when nutritionally superior patches were readily available and in spite of the expected costs owing to larval competition, we wished to assess whether females would moderate their strong tendency to copy when the occupied patches either contain numerous larvae or have already experienced heavy consumption by larvae. Finally, to assess possible informational benefits to females, we tested whether larvae were better than adult females at assessing food quality.

## Materials and Methods

### Nutritional Titration

**Figure 1:**
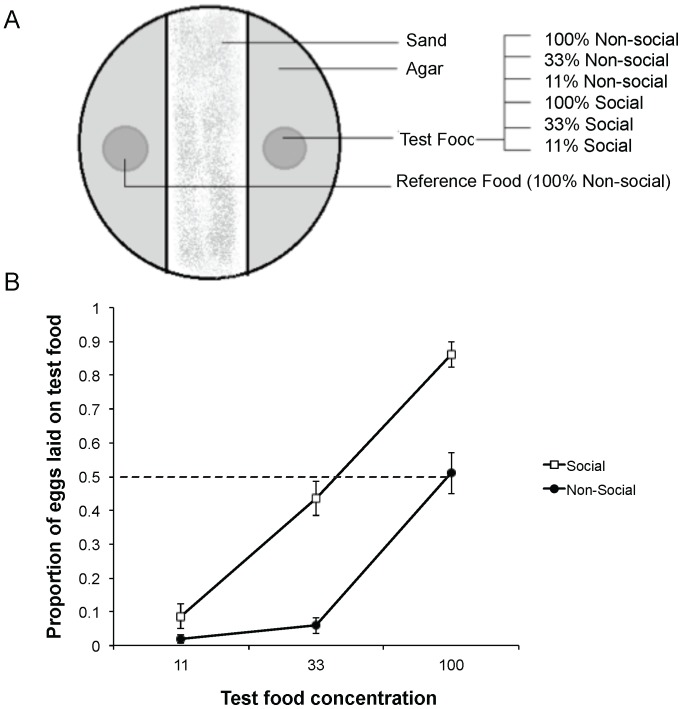
Nutritional titration. (A) Each dish always contained a reference disc and one of six types of test discs varying in nutritional concentration and larval presence. Sand at the centre of the dish prevented larval crawling to the reference food. (B) The average proportion of eggs (±1 SE) laid on the test disc as a function of its nutrient concentration and presence or absence of larvae (social or non-social). The horizontal dashed line indicates random choice. N = 30 replicates per treatment. Females laid more eggs on the test food in the presence than absence of larvae.

We maintained two population cages of several hundred *Drosophila melanogaster Canton-S* following standard protocol [27]. To quantify the value that females assign to patches already occupied by larvae, we placed each of 192 recently mated female inside a 60 mm Petri dish. The bottom of the dish contained agar, which provided moisture. On top of the agar, we placed two discs cut from a thin layer of fly medium. Both discs were 1.1 cm in diameter and each contained 0.5 ml food (Fig. 1a). The reference disc always had standard food in which 1 litre contained 60 g dextrose, 30 g sucrose, 32 g yeast, 75 g cornmeal, 20 g agar, 2 g methyl-paraben and water. The test disc was either fresh (non-social) or contained five early second instar larvae that had fed on that disc for 24 h (social). The test disc had standard food or one of two lower food concentrations containing either 33% or 11% of the nutrients (dextrose, sucrose, yeast, and cornmeal) available in the standard food and a larger proportion of water. The reference and test discs were 3 cm apart with the central 2 cm being a trough filled with fine sand (Fig. 1a) to prevent larvae located on the social discs from crossing to the reference discs. We housed all dishes in a chamber kept at 25°C and 90% RH and allowed the females in the Petri dishes to lay eggs overnight for 14 h. Then we discarded the females and counted the number of eggs laid on each disc. We used a generalized linear model with a Tweedie distribution and identity link function and conducted pairwise comparisons with Bonferroni corrections and 95% Wald confidence intervals [36]. See Data S1 for the raw data for all experiments.

### Larval Success on Social vs. Non-Social Food

Our nutritional titration experiment indicated that females perceive social food with about one third the nutrients as equally valuable as the non-social reference food (Fig. 1b). We thus wished to quantify the success of females' eggs on social vs non-social food discs of distinct nutritional concentrations. To assess the value of laying eggs on currently versus previously occupied patches, we also included a previously social treatment. We had a total of 6 treatments involving 2 food concentrations, 100% and 33%, and 3 social treatments, non-social, social and previously social. We omitted the 11% food concentration because females in the titration experiment mostly avoided it even when it was social (Fig. 1b). The food discs were identical in constitution and volume to the 100% and 33% food discs in the titration experiment.

The non-social discs contained unmodified food. To generate the social and previously social discs, we placed on each disc 5 24-hour old first instar larvae and allowed these larvae to feed for 24 hours. In the social disc treatment, we kept the now second instar, 48 h old larvae on each disc. In the formerly social disc, we removed the larvae. That is, both the social and previously social discs were equally modified by the five larvae prior to the placement of focal eggs. Then the focal larvae emerging on the formerly social disc could reap potential benefits from such previous food modification without experiencing competition with the older larvae. Thus the formerly social disc gave us a greater power for quantifying possible benefits of prior food modification by larvae.

We placed each food disc inside a 35 mm Petri dish lined with agar, added to each disc five focal eggs and housed all the dishes in a chamber kept at 25°C and 90% RH. When the five older larvae in the social dishes pupated, we removed these pupae. We then monitored the number of focal larvae reaching pupation and calculated the larval developmental rate as the cumulative proportion of larvae reaching pupation while taking the final pupal number as 1. We counted all eclosing adults and calculated the proportion of eggs that produced adults. Because females are heavier than males, we sexed the adults, dried them in an oven at 70°C for 3 days and weighed groups of five flies of the same sex on a microbalance.

Because no larvae survived in the social 33% treatment, we conducted two separate analyses. First, we omitted the social treatment and compared larval performance in the four treatments of non-social and formerly social on 33% and 100% food. Second, we compared larval performance in all three treatments of non-social, formerly social and social on the 100% food.

**Figure 2:**
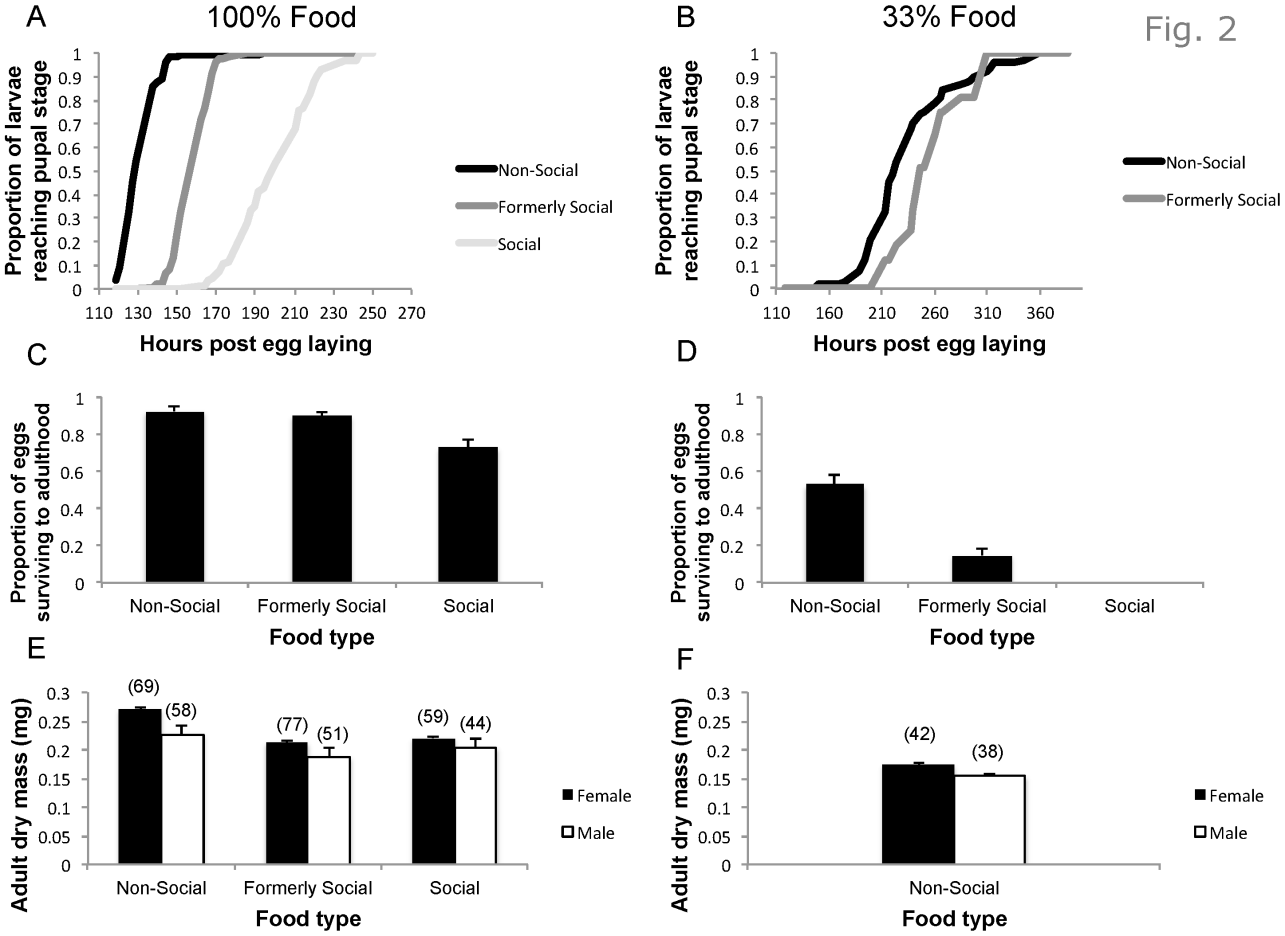
Larval performance as function of a disc's nutritional and social status. The left panels (A, C, and E) refer to the 100% nutrients while the right panels (B, D, and F) refer to the 33% nutrients. (A) and (B) show the time it takes for the larvae to develop from eggs into pupae. (C) and (D) show the proportions of eggs that survived to adulthood (mean+SE). In (B) and (D), survival in the social treatment was 0. (E) and (F) show the adult dry mass (mean+SE). N =  30 replicates for each treatment. The number of eclosing adults is shown above the bars in panels E and F.

We analyzed larval development rate and the proportion of eggs surviving to adulthood using a generalized estimating equation with a gamma distribution and log link function [29]. We had sufficient sample sizes for analyzing adult dry mass only for the 100% food (Fig. 2E, F). These data met ANOVA assumptions and we thus used a two-way ANOVA with a Tukey HSD. We conducted all post-hoc pairwise comparisons using the sequential Bonferroni method adjusting for multiple comparisons.

### Larval Success on Abundant Food

In our previous larval success experiment, larvae were reared on 0.5 ml of food. Because the results indicated strong effects of competition, we tested larval success on social and non-social discs each containing 2.5 ml of 100% food. As a reference, fruit fly laboratories typically rear a few dozen flies per vial containing 5 ml of similar food [37], [38]. By providing abundant food, we wished to maximize our ability to detect possible benefits that larvae may gain from developing on social food. All other protocol details were similar to those detailed above. That is, The social food contained 5 larvae and the non-social food had no larvae.

### Females' Patch Choice When the Social Patches Have Had High Larval Densities

**Figure 3:**
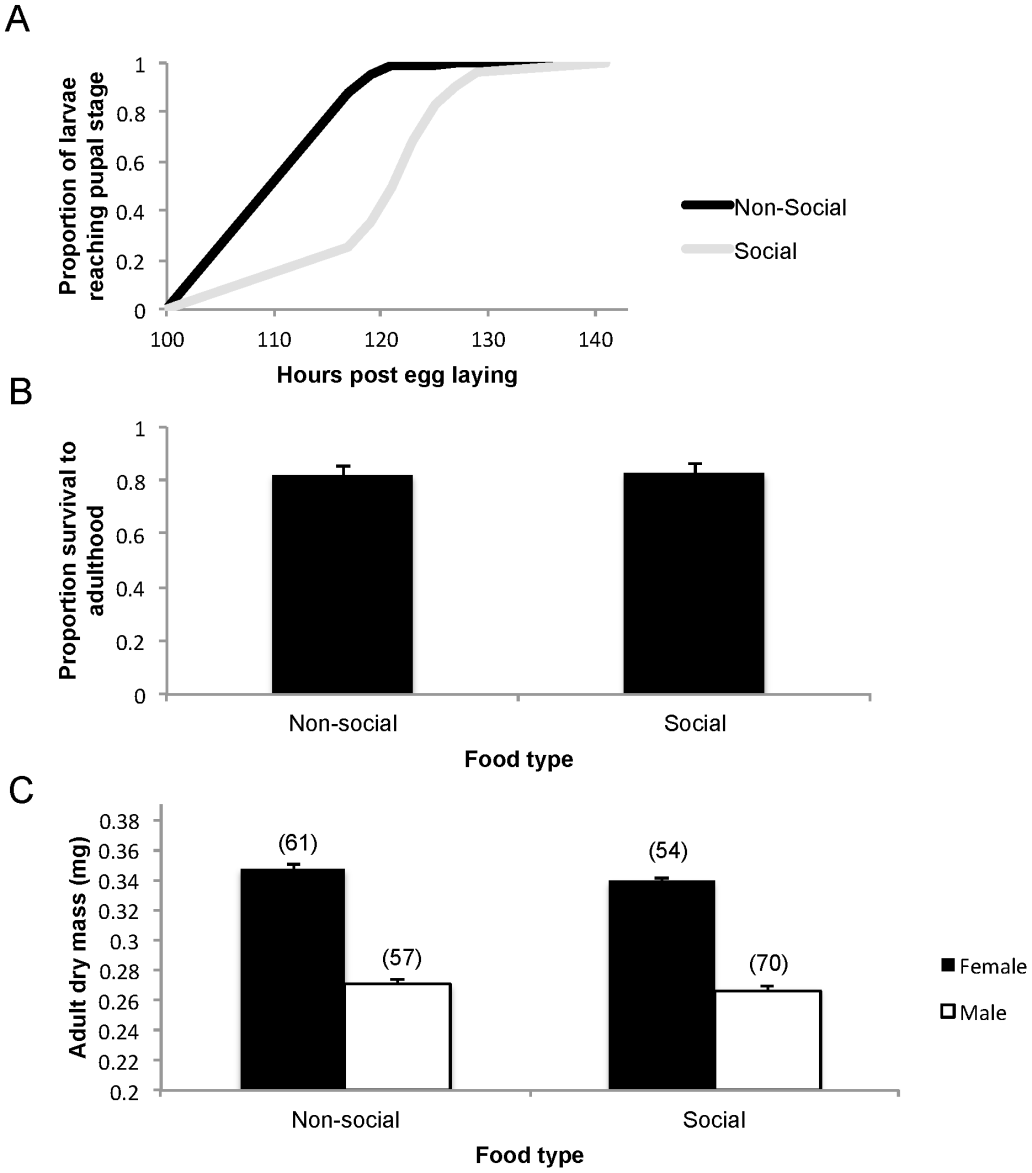
Performance measures of focal larvae on abundant food. Discs were either social or non-social (n = 30 replicates per treatment). (A) Time from egg laying to pupal formation (B) The proportion of eggs surviving to adulthood (mean+SE). (C) The adult dry mass of females and males in both conditions (mean+SE). Numbers in brackets above the bars indicate the number of adults in each group.

In our titration experiment (Fig. 1B), females showed a strong preference for laying eggs near larvae even though this reduced their offspring success in our laboratory settings (Figs 2, 3). Because larval crowding and the consequent lower larval success are prevalent in nature as well [39], [40], we expected females to make egg laying decisions that balance their perceived benefit from laying next to larvae versus the expected cost due to larval overcrowding. We thus allowed females to choose between either a non-social patch and a social patch occupied by 5 larvae, or a non-social patch and a social patch occupied by 20 larvae. We predicted that females would lay a lower proportion of eggs on the social food when it was more crowded.

We used a protocol modified from Durisko et al [30]. We placed each recently mated female inside a plastic cage (15 cm wide, 30 cm long, and 15 cm high), which contained two 35 mm Petri dishes placed at the opposite far corners of each cage. One dish was non-social and the other was social. Both dishes contained 0.5 ml food discs composed of 100% standard lab diet. The dishes were lined with a layer of agar to prevent desiccation. Non-social food discs were unoccupied. The social food discs had either 5 or 20 middle second instar larvae, which we had added 6 h before the addition of females.

We allowed the females to lay eggs overnight. In the following morning, we removed the females from the cages, counted the number of eggs on each food disc and analyzed the proportion of eggs laid in the social dish out of the total number of eggs that a female laid. Based on preliminary data indicating effects of larval density on egg location, we also counted the number of eggs laid on the agar layer within 1 cm of the food disc and calculated the proportion of eggs laid on agar versus food in the social dish. We analyzed the data using a generalized linear model with a Tweedie distribution and log link function.

The experiment above tested females' sensitivity to larval density. It is possible however, that females are more sensitive to the condition of food as indicated by the microbial community and waste products rather than to the number of larvae already on the food. To test this possibility, we allowed females to choose between either a non-social patch and a social patch that had been previously occupied by 5 larvae, or a non-social patch and a social food patch that had been previously occupied by 20 larvae. Again, we predicted that females would lay a lower proportion of eggs on the social food that had been more crowded.

Forty-eight hours before the experiment, we transferred groups of either 5 or 20 middle second instar larvae to social food discs and kept them in 35 mm Petri dishes lined with agar. We also kept unoccupied food discs in Petri dishes lined with agar. All food discs contained 0.5 ml of 100% standard lab diet. By the day of the experiment, all larvae on the social discs had pupated. We then placed one non-social food disc and one social disc in 60 mm Petri dishes lined with agar. The social disc had been previously consumed by either 5 or 20 larvae but was free of larvae and pupae by the time of the test. Discs were placed 2 cm apart. We then added a recently mated female to each 60 mm dish through a hole in the lid, which was then plugged with foam. We allowed the females to lay eggs overnight. In the morning, we removed the females from the dishes and counted the number of eggs on the social and non-social food discs. We analyzed the proportion of eggs on each type of social food using a generalized linear model with a Tweedie distribution and log link function.

### Adult vs. Larval Abilities to Detect Differences in Yeast Concentration of Food

Because we documented a lower larval success of eggs laid at social patches, we wished to test whether the benefit of patch choice copying is related to information rather than to joining. To this end, we tested whether larvae could detect pertinent patch characteristics that adult females could not. We had two treatments testing larval and adult females' abilities to detect differences in yeast content between adjacent patches. We focused on yeast rather than sugar because larval and adult perception of sweetness is well documented [41]–[44]. One test involved a reference 100% standard fly medium vs standard medium with only 33% of the yeast content, and the other test involved a reference 100% standard medium vs standard medium with only 50% of the yeast content. All other medium ingredients were identical.

We added either one recently mated adult female or five mid-second instar larvae to Petri dishes containing one reference food disc and one food disc with lower yeast concentration (either 33% or 50%) placed 2 cm apart. We added the adults and focal larvae in the evening at an identical location 1 cm between the food discs. We gave them 14 hours to decide where to lay eggs or feed. In the following morning, we counted the number of eggs laid on each food disc in the adult female treatments and counted the number of larvae on each food disc in the larval treatments. We then calculated the proportion of eggs laid and the proportion of larvae on the reference 100% disc and analyzed the data with a generalized linear model with a Tweedie distribution and identity link function.

## Results

### Nutritional Titration

Females laid significantly higher proportions of eggs on the test food when it was social than non-social at all three food concentrations (Wald χ^2^_1_ = 49, P<0.001 for the main effect and P<0.01 for the three pairwise comparisons with Bonferroni corrections, Fig. 1).

### Larval Success on Social vs. Non-Social Food

#### Larval performance across food qualities

Owing to 100% mortality in the social 33% food treatment, we could compare larval performance across food qualities only for the non-social and previously social treatments. Larvae developed much faster (Wald χ^2^_1_ = 474.74, P<0.001; Fig. 2A,B) and had higher survival rates on the 100% than 33% food (Wald χ^2^_1_ = 75.6, P<0.001; Fig. 2C,D). Similarly, larvae developed much faster (Wald χ^2^_1_ = 33.361, P<0.001; Fig. 2A,B) and had higher survival rates in the non social than formerly social treatments (Wald χ^2^_1_ = 75.769, P<0.001; Fig. 2C,D).

Because survival rates in the 33% food treatment were low, we could only compare adult body mass across food qualities in the non-social treatments. Adults in the 100% food quality were much heavier than those in the 33% food quality (Wald χ^2^_1_ = 512.96, P<0.001; Fig. 2E,F).

#### Larval performance across social treatments

This analysis could include only the 100% food owing to 100% mortality in the social 33% food treatment. Larvae developed significantly faster in the non-social treatment, intermediate in the formerly social treatment, and slowest in the social treatment (Wald χ^2^_2_ = 1700, P<0.001; Fig. 2A). Post-hoc pairwise comparisons showed that each treatment was significantly different from the other two (P<0.001).

Survival to adulthood was significantly affected by the social treatment (Wald χ^2^_2_ = 13.9, P = 0.001; Fig. 2C). Survival was similar in the non-social and formerly social treatment (post-hoc pairwise comparison, P = 0.709) but higher in each of these treatments than in the social treatment (post-hoc pairwise comparisons, P = 0.002 and 0.005 for the non-social and formerly social treatment respectively).

Adult mass was significantly affected by the social treatment (F_2,61_ = 85.2, P<0.001; Fig. 2E). In both males and females, adults of the non social treatment were heavier than those of the social and formerly social treatments (Tukey HSD, P<0.001). While males of the formerly social treatment were lighter than those in the social treatment (P = 0.007), females of the formerly social and social treatments had similar masses (P = 0.438).

### Larval Success on Abundant Food

Larvae developed faster in the non-social condition than in the social condition (Wald χ^2^_1_ = 34.683, P<0.001; Fig. 3A). However, the same proportion of focal eggs survived to adulthood (Wald χ^2^_1_ = 0.014, P = 0.905; Fig. 3B). Adult flies in the non-social condition were heavier than adults in the social condition (Wald χ^2^_1_ = 4.515, P = 0.034; Fig. 3C).

### Females' Patch Choice When the Social Patches Have Had High Larval Densities

**Figure 4:**
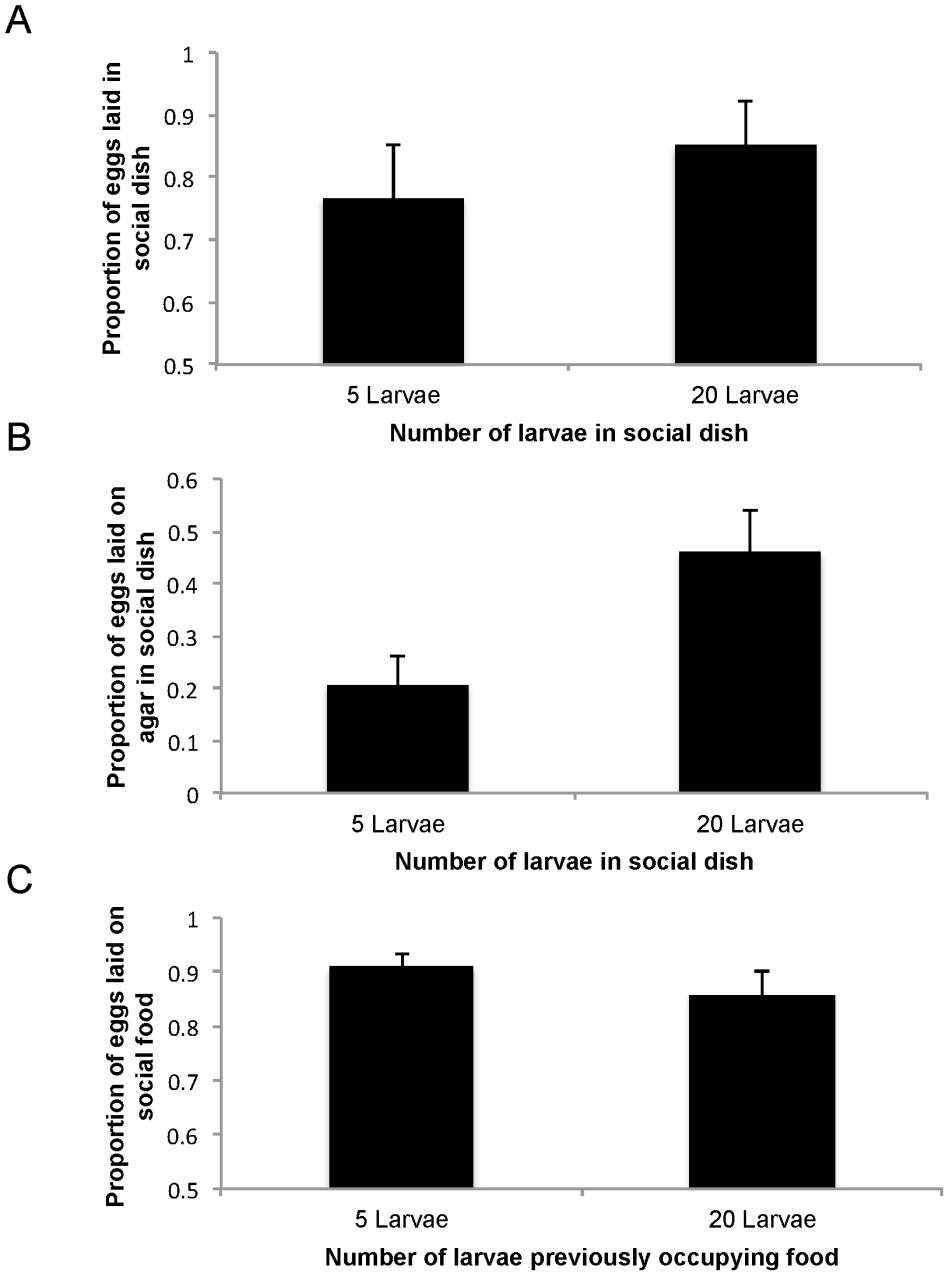
Social patch choice under high larval densities. The proportion (mean+SE) of eggs laid at the social disc, which currently (A, B) or previously (C) contained either five or 20 larvae. In each case, females could choose between laying at a social or non-social disc. (A) The proportion of eggs laid in the social dish out of all eggs laid. (B) The proportion of eggs laid on agar rather than on the food disc out of the eggs laid in the social dish. N = 24 replicates per treatment. (C) The proportion of eggs laid on the social disc, which had been previously consumed by either 5 or 20 larvae, out of all eggs laid. No larvae were present on the food at the time of egg laying. N = 28 replicates per treatment.

Females laid similar proportions of eggs in the social dishes occupied by 5 and 20 larvae (Wald χ^2^_1_ = 0.204, P = 0.651; Fig. 4A). However, females placed a greater proportion of their eggs on the agar in the social dishes with 20 than 5 larvae (Wald χ^2^_1_ = 4.649, P = 0.031; Fig. 4B). When females had a choice between non-social and previously occupied social discs, they laid a similar proportion of their eggs on the social disc regardless of the number of larvae that had previously occupied it (Wald χ^2^_1_ = 0.472, P = 0.492; Fig. 4C).

### Adult vs. Larval Abilities to Detect Differences in Yeast Concentration Of Food

**Figure 5:**
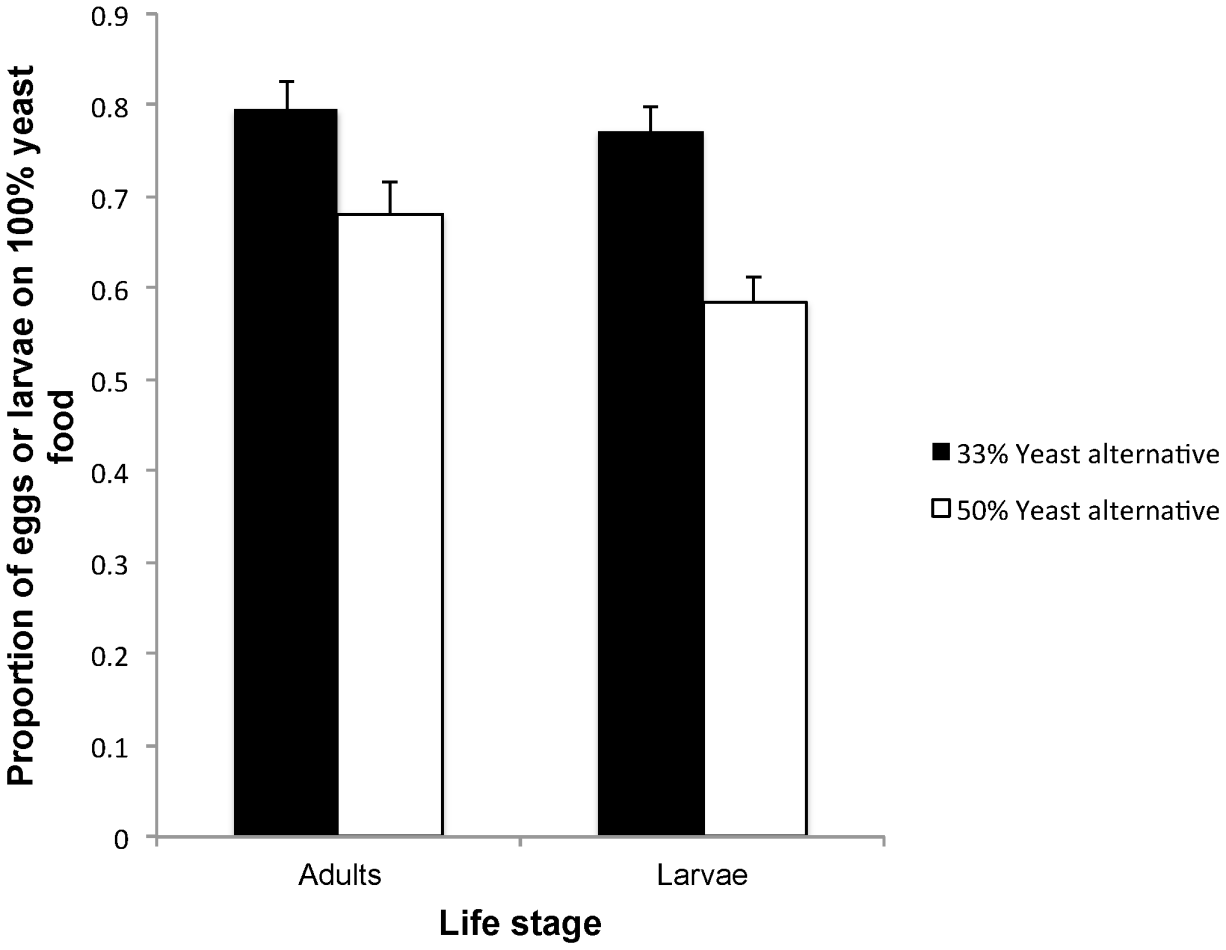
Patch choice by adult females versus larvae. In one experiment (black bars), adult females or larvae had a choice between a disc containing the regular yeast concentration (100%) or a disc containing 33% of the regular yeast concentration. In the other experiment (white bars), adult females or larvae had a choice between a disc containing the regular yeast concentration (100%) or a disc containing 50% of the regular yeast concentration. N = 80 replicates per nutrition treatment for larvae, and N = 60 replicates per nutrition treatment for the adult females.

The proportion of eggs that females laid on the 100% food and the proportion of larvae choosing the 100% food were similar when the alternative had only 33% of yeast concentration (Wald χ^2^_1_ = 0.227, P = 0.634; Fig. 5). When the alternative was 50% yeast concentration, females showed a greater preference than larvae for the higher quality food (Wald χ^2^_1_ = 3.835, P = 0.05; Fig. 5).

## Discussion

Our titration experiment (Fig. 1) indicated that, while females were highly sensitive to the nutritional values of alternative patches, they perceived low-nutrition patches occupied by larvae (social patches with 33% of the nutrients) as suitable as the reference, unoccupied patches (non-social patches with 100% of the nutrients). The larval success experiment (Fig. 2) indicated that the females' sensitivity to nutrient concentration was highly justified: their larvae developed significantly faster, had higher survival rates and produced larger adults on the non-social 100% than non-social 33% patches. Because females were willing to trade the nutritional quality of patches for the opportunity to lay eggs at patches already occupied by larvae, we expected that such choice would translate into some larval benefit. However, we did not find such an advantage. First, in all cases, larval success on the social patches was lower than that on non-social patches (Fig. 2). Second, in the previously social treatment, we removed the larvae that had occupied the patches before placing focal eggs. This allowed us to test for possible benefits that females could gain from laying eggs at patches that have been occupied by larvae while eliminating the negative effects of competition from such larvae. Even in this case, however, we found a cost rather than benefit from laying on previously occupied patches (Fig. 2). Finally, one could argue that our larval to food-volume ratio was too high so that larval competition obscured a gain occurring when food is abundant. To address this possibility, we repeated the larval success experiment with a much lower larval to food-volume ratio. Even in this case, however, larvae performed better under the non-social then social treatment (Fig. 3). The mechanism underlying this negative social effect is unknown and will require close examination in the future.

To further assess the egg laying decisions by females, we wished to quantify females' responses to clear signs of competition in social patches due to either the previous or current presence of many larvae. Although we expected females to reduce their preferences for the social patches when they were either crowded or heavily exploited, we found no such moderation (Fig. 4). Finally, although the sense of taste provides important information about the nutritional quality of food, it is insufficient for assessing whether all nutrients required for optimal larval development are available [41], [43]–[45]. We thus proposed that the presence of feeding larvae is the best cue indicating to females that a substrate is nutritionally sufficient. First, the substrate is adequate for sustaining the larvae as indicated by the fact that they are alive. Second, the larvae are highly mobile and are adept at exploring and settling at the best locally available food [29], [46]. Contrary to our expectation, however, we found in two experiments that adult females were as sensitive as larvae to realistic variations in nutritional qualities (Fig. 5).

To summarize our key results, we have strong evidence that females assign high values to patches already occupied by larvae as we quantified by titrating the nutritional quality of the patches (Fig. 1) and we could translate these values into the relevant currency of larval success (Figs 2, 3). Our data, however, indicated neither informational gain (Fig. 5) nor direct benefits from patch choice copying (Figs 2, 3). How can this puzzle be resolved? We propose four non-mutually exclusive explanations related to fruit flies' ecology under natural settings. The first three explanations deal with microbial ecology while the last one focuses on fruit fly parasitoids, which, alongside microbes, are the prominent natural enemies of fruit fly larvae. While the third explanation (microbial information) pertains to the informational benefits of patch choice copying, all other three explanations relate to the direct benefits to larvae from joining other larvae.

### Competition with Microbes

While fruit flies feed on yeast species growing on fallen fruit [47], such fruit are also consumed by numerous other fungi as well as bacteria. This means that the other microbes can adversely impact yeast through exploitation competition. Furthermore, microbial interference competition involves a rich arsenal of compounds toxic to other microbes as well as to animals. That is, such compounds can either hamper yeast growth, thus reducing the amount of food available to larvae, or have direct negative effects on larval survival and growth [48]–[53]. Although highly pertinent for our understanding of the behavior of larval and adult fruit flies, the microbial ecology relevant to fruit flies remains mostly unexplored. A notable exception is work by Rohlfs and colleagues [54], [55], which quantified negative effects of three mold species on fruit fly larvae and indicated that groups of five and 10 larvae were more effective at suppressing mold growth than single larvae. Another relevant observation is that fruit flies possess a dedicated olfactory circuit tuned to geosmin. Fruit flies rely on this circuit to avoid feeding and egg laying on substrates containing geosmin-producing microbes, which are harmful to fruit flies [56]. This indicates that fruit flies are sensitive to the constitution of microbial communities at prospective egg laying sites. It is thus likely that, by preferring to lay eggs at patches already occupied by larvae over unoccupied patches, females in natural settings ensure that their newly hatched larvae will be better protected from microbes harmful either to their larvae or to their larval yeast-food.

### Group Enhancement of Favourable Yeasts

There appear to be mutualistic interactions between some yeast species and fruit flies. Adults and larvae inoculate fruit with yeast and larval activity promotes the growth of certain yeast species [57]–[59]. While some of the positive effects of larvae on yeast can be modulated through churning of the substrate, the larval gut bacteria also produce antifungals, which could selectively suppress mold and thus enhance the growth of the preferred yeast food [31], [60]–[62]. Intriguingly, adult and larval fruit fly attraction to food inhabited by larvae is mediated by volatiles emitted from gut bacteria [31]. Hence it is likely that females in nature lay eggs in occupied patches because such patches are more favourable for further growth of yeast food than are unoccupied patches.

### Microbial Information

While we found no evidence that larval presence provides superior nutritional information about patch quality that females cannot readily assess, the discussion above suggests that larval presence is the best indicator that the microbial ecology is favourable to larval growth. That is, it is likely that different fruit patches allow for the optimal growth of different microbial species with only some of them being hospitable to fruit flies. For example, substrates may vary in their ability to sustain the growth of harmful mold and bacteria versus the yeast species favoured by fruit flies. Assuming that females cannot assess all the relevant ecological settings that would influence fungal growth, the presence of thriving larvae may be the best cue indicating that a patch is providing the appropriate microbial environment.

### Parasitoid Avoidance

Larval parasitoids are a major source of fruit fly mortality in natural settings and fruit flies possess a suite of behavioral and physiological adaptations for reducing parasitoid success [63]–[66]. One way by which larvae can avoid parasitism is through hiding in micro-sites inaccessible to parasitoids. Although newly hatched larvae are not proficient at burrowing, older larvae, especially ones in the third instar stage, have stronger and larger mandibular hooks containing several teeth [67] and they spend much of their time tunnelling deep inside the substrate [34]. It is thus possible that, by laying eggs close to larvae, females ensure that their hatching offspring can hide in burrows dug by the older larvae. Limited evidence indeed indicates that larvae hidden deep in natural fruit experience lower rates of parasitoid attacks [24].

### Patch Choice Copying in Other Species

Our work on the value of patch choice copying in fruit flies can inform and be informed by research on copying in other species. Perhaps the best studied and most relevant system involves the economically important bark beetles (Scolytidae), which aggregate at host trees. While there are many species of bark beetles, we focus here on obligate parasites, which attack and kill trees [68]. Long-distance attraction to host trees in bark beetles is mediated by pheromones. Early colonizers benefit from attracting others because a critical mass of beetles and perhaps associated fungi are necessary for overcoming the massive defence mounted by the host tree [68]–[70]. Because prospective females gain from joining patch occupiers, the adaptive function of patch choice copying is clear.

There are at least two major differences between the fruit fly and bark beetle systems. First, in the bark beetles, there is active recruitment by early colonizers, which is crucial for their success [69], [71]. In fruit flies, cis-vaccenyl acetate (cVA), has been referred to as an aggregation pheromone [57], [72]. However, cVA is produced only by males, who transfer it during copulation to females [73], in which it signals to prospective males that the females are recently mated and unreceptive. Indeed females emitting cVA are much less attractive to males than females with no cVA [74]–[76]. It is thus likely that cVA has a relatively negligible role in long-distance attraction compared to the dominant role of microbial volatiles [31], [77], [78]. That is, there is no critical evidence indicating active recruitment of conspecifics in fruit flies.

The second and somewhat related difference between the bark beetle and fruit fly systems is the change in patch attractiveness with density. In the bark beetle system, there is a clear decline in tree attractiveness once a threshold beetle density has been reached. Such decline can readily be explained. Functionally, the occupiers no longer require further individuals once the tree is dying. Mechanistically, the occupiers can readily modulate patch attractiveness by ceasing to emit the aggregation pheromone [69], [71]. In the fruit fly system, we failed to identify the predicted lower patch attractiveness under higher density. It is likely, however, that, in natural settings, cues from microbes associated with high density could decrease patch attractiveness or even repel females, as does geosmin discussed above.

Most other systems in which patch choice copying occurs are not as well studied as bark beetles. We suggest, however, that fruit flies can serve as an excellent general model system for further research on the topic owing to their amenability to research in the ecological, evolutionary and mechanistic domains. Our work so far suggests that direct benefits from joining others are likely in many systems even when such benefits are not observed under controlled settings. The most likely reason for such discrepancies is an involvement of harmful microbes in natural settings, which a group is more likely to overcome than an individual. Similarly, because the microbial ecology and dynamics is complex, prospective individuals probably gain the best available information from relying on others, because the others' presence indicates a suitable microbial setting. Our proposition about the central importance of microbes will require extensive experimental work in collaboration with microbial ecologists.

## Supporting Information

Data S1 **The raw data for the information shown in the figures.**
(XLSX)
